# Metacognitive regulation: emergence, focus, and function in interprofessional collaborative learning

**DOI:** 10.1007/s10459-025-10458-z

**Published:** 2025-07-28

**Authors:** Erika Österholm, Tuike Iiskala, Reetta Mustonen, Mari Murtonen

**Affiliations:** 1 Department of Teacher Education, University of Turku, Turku, 20014 Finland; 2 Department of Nursing Science, University of Turku, Turku, 20014 Finland

**Keywords:** Interprofessional collaborative learning, Metacognitive regulation, Socially shared metacognitive regulation, Health and social care education

## Abstract

Effective learning depends on metacognitive regulation (MR), especially in interprofessional learning (IPL) contexts, which typically involve collaboration on diverse cases. However, education research and support have devoted insufficient attention to the regulation of group collaborative learning. The present study employed rigorous socio-cognitive content analysis to examine IPL in small mixed groups (*N* = 7) of undergraduate health professionals (*N* = 47). A four-week online group discussion period was used to explore the emergence, focus, and function of MR and socially shared metacognitive regulation (SSMR). The results confirm the emergence of MR in every group, ranging from 15 to 25% of sentences produced regarding four themes: task production, case content, interprofessional content, and group collaborative learning. In every group, MR focused more on high-level content processing than on low-level task production. The findings indicate that the primary function of MR is to monitor learning, with little planning or evaluation. While most MR was socially shared, there were clear differences between the groups in this regard. Overall, statistical testing revealed significant differences between the seven groups in terms of the emergence, focus, and function of MR and the extent of SSMR. It can be concluded that consistent high-level collaboration demands explicit educational support to amplify MR emergence, with a greater focus on content integration, planning and evaluation. The study augments the limited existing literature on the role of MR and SSMR in interprofessional collaborative learning; it shows how MR in collaborative learning can enhance instruction in health and social care education contexts.

## Introduction

Interprofessional collaborative learning seeks to benefit from the diverse knowledge and skills of a group’s various members. Collaboration is one of the declared core competences of interprofessional education (IPE) (IPEC, 2023), and collaborative learning is considered central to interprofessional learning (IPL) (Bogossian et al., 2023). To fully exploit the benefits of IPL, strong collaborative learning is imperative. However, not all group work is effective in this regard (Summers & Volet, 2010); for example, some students are known to lack collaborative learning skills, which involve more than exchanging information. According to Wilhelmsson et al. (2012, p. 90), “simply letting students work *in* teams does not necessarily mean they are actually working *as* a team.”

Collaborative learning aims to trigger learning and produce the desired effects through interaction (Dillenbourg, 1999). In high-level collaboration, the aim is to jointly create knowledge and a deep understanding of the subject matter (Summers & Volet, 2010). To that end, collaborative learning tasks must be relevant and should promote interdependence within the group (Scager et al., 2016). Smeets et al. (2024) demonstrated that this kind of interdependent interprofessional collaboration is deficient in health and social care education settings.

To improve interprofessional collaborative learning, interprofessional knowledge must be integrated rather than merely being the sum of group members’ ideas. For interprofessional experts, metacognitive abilities matter (Boon et al., 2019) because collaborative problem solving across diverse patient cases depends in part on metacognitive “thinking about thinking” (Miller et al., 1970). It follows that metacognitive regulation (MR) plays an essential role in supporting quality learning for collaborative healthcare (Bransen et al., 2022). Volet et al. (2009) argued that if we are to understand productive collaborative learning, we must examine actual group learning activities, including interdependent knowledge construction and social regulation, i.e., the socially shared metacognitive regulation (SSMR).

In health and social care education settings, these key socio-cognitive, egalitarian, and partnership aspects of collaborative learning (Vauras et al., 2003) clearly warrant closer attention. To consolidate high-level collaborative learning in IPE, it seems essential to forge theoretical connections that narrow the research gap between MR and IPL. As Dillenbourg (1999) put it, we must “zoom in” to study collaborative interactions more closely in order to build this new understanding.

### Metacognitive regulation and socially shared metacognitive regulation

Metacognition has been studied since the 1970s (Brown, 1982; Flavell, 1979), first at the individual level and later in social learning contexts as a social phenomenon (see e.g., Garrison & Akyol, 2015; Volet et al., 2013). The metacognitive strategies employed by the student´s to regulate their learning processes are grounded in the concept of self-regulated learning (SRL) (Pintrich, 1999). Regulation encompasses three distinctive processes or models of metacognitive control, specifically planning, monitoring, and evaluating. Planning involves goal setting, choosing cognitive learning strategies and reviewing resources; monitoring refers to being aware of students’ individual or collective comprehension of a given task; and evaluating relates to assessment of the targeted task performance or skill construction compared to given standard (Pintrich, 1999; Schraw & Moshman, 1995; Zimmerman, 1986, 2002). While the research on SRL focuses to describe learners´ deliberate regulation of cognitive, behavioral, motivational and emotional processes (Hadwin et al., 2018; Järvelä & Miller, 2013), the current study examines metacognitive regulation as a joint cognitive and social process.

In general, in collaborative learning, MR refers to a group´s ongoing monitoring of their learning to check learning progress in pursuit of joint or shared learning goals (Schoor et al., 2015). A number of previous studies have confirmed the role of MR in collaborative learning (e.g., Iiskala et al., 2011, 2015; Khosa & Volet, 2014; Volet et al., 2009). Prior research has shown how the active regulation of cognitive processes impacts learning and supports deep learning both at the individual level and during collaborative learning (see for example Biggs, 1988; Vermunt & Vermetten, 2004: Volet et al., 2013).

In cognitive interactions during so-called *low-level* processing, a group shares information without evidence of knowledge integration. In contrast, during *high-level* processing, group members provide justifications, ask questions, and negotiate the learning content. High-level content regulation in particular leads to the collaborative creation of meaningful new knowledge (Volet et al., 2009). Iiskala et al. (2011) and Khosa and Volet (2014) suggested that regulation of content and understanding are central opposed to task production regulation. The present study examines how MR emerges as low-level task production and high-level content management. In particular, we looked at how low-level MR regulates learning in terms of task production and organisation while high-level MR focuses on content problem solving, coordination, and justification.

Recent studies of MR have looked at the phenomenon of socially shared metacognitive regulation (SSMR) in collaborative learning, where the “metacognitive process is shared between the participants” (Iiskala, 2015, 17)—that is, at least two participants engage in mutual and reciprocal cognitive regulation of the group learning process and truly shared by participants (e.g., Liskala et al., 2004; Iiskala et al., 2015; Khosa & Volet, 2014). Thus, SSMR is not just a sum of individual regulatory acts. For present purposes, we employ the term SSMR as in Schoor et al.’s (2015) theory-based review of regulative processes, in which they state that “the term *socially shared metacognition* emphasizes that it is cognition that is regulated” (p. 107). As IPL exploits group situations to benefit from the knowledge and skills of different professions and disciplines, SSMR is especially important in interprofessional contexts.

IPL and MR have been studied extensively but rarely together. Some previous studies have explored self- and co-regulation in interprofessional online learning (Zheng et al., 2023); self-, co-, and socially shared regulation (Bransen et al., 2022); metacognitive models in designing IPE curricula (Wilhelmsson et al., 2012); metacognitive skills in IPE (Poirier et al., 2017); and metacognitive awareness (Lovell et al., 2021). However, we still know very little about MR in interprofessional collaborative group processing contexts, including how much is regulated, what (focus) is regulated, and how focus relates to the various functions of MR. To our knowledge, the topic of SSMR in interprofessional collaborative learning settings remains unexplored. Social regulation is of particular interest in IPL contexts, and MR and SSMR are core collaborative competences for every health professional.

### Study aims

Given the lack of previous research on the metacognitive regulation of learning in interprofessional groups, we decided to explore the emergence, focus, and function of MR and SSMR during small-group discussions of patient case assignments. An enhanced understanding of how collaborative learning processes are regulated will enable educators to develop more effective IPE for learning based on interprofessional knowledge and skills and group interactions.

### Research questions

1. To what extent does metacognitive regulation (MR) emerge in interprofessional group learning?
2. What is the observed focus and function of the emergent MR? How is focus distributed in low- and high-level MR?
3. To what extent does socially shared metacognitive regulation (SSMR) emerge in such contexts?

## Method

Previous studies have emphasized the need for sensitive but rigorous methods for MR analysis (Volet et al., 2013; Khosa & Volet, 2014). For the purposes of the present study, we employed qualitative content analysis (Hsieh & Shannon, 2005) to analyse written data from asynchronous group discussions. Based on these data, we investigated the emergence of MR and SSMR during interprofessional collaborative learning in a higher education setting. Coding and analyses were completed in several phases and in a stepwise manner to explore MR as a dynamic multilevel phenomenon. Following the content analysis, MR and SSMR were quantified to analyse similarities and differences between the groups. The aim was to provide a more in-depth comparison of the results between the groups. Using SPSS 29, statistical analyses including crosstabulation and z-testing were performed to explore connections between the groups.

### Participants and study context

The data comprised messages (comments) produced by interprofessional small groups (*N* = 7) in a computer-supported collaborative learning environment. Students (*n* = 47; 87% female, and 13% male) addressed the case of a neurological patient over four weeks of an interprofessional course. Sampling was purposive. Each small group included 6 or 7 students from the disciplines of medicine, occupational therapy, physiotherapy, psychology, social science/services, and speech therapy. In the absence of a medical student, there was double representation of another discipline in some groups. Participants had completed at least two years of their own degree program, and all students on the course could join the study. Before commencing, written consent (including the collection of discussion data) was individually secured from the 84% who agreed to participate.

#### Asynchronous small group discussions

The main goal of the course (3–5 ECTS) was to provide a learning platform for interprofessional collaboration. The learning outcomes aimed to enhance skills to understand the needs of the patient, the role of different professions, applying their own professional skills in interprofessional group discussion and developing both interaction and consultation skills. Initial lectures were followed by four weeks of group discussion and (finally) case seminars. The lectures sought to orient students to the key elements of interprofessional work. The group discussions then aimed to develop an interprofessional evaluation and rehabilitation plan for the (imaginary) case of a clinical neurology patient and case seminars provided a forum to review each case with teachers and students. The cases were developed by the teachers and coordinators responsible for the course. The educators aimed to create equally challenging case assignments and drew on their solid expertise of interprofessional work. There were four cases and two groups worked on the same case: “*a pupil with learning challenges”*, for groups A and C; “*child with oral fissure”*, for groups B and F; “*an elderly memory patient*”, for group D and “*an adult customer with brain injury*”, for groups E and G. Each group had its own closed discussion area; participants were instructed to participate actively, reflect on the case together and write their thoughts as comments, providing justifications, proposing alternative views, and asking for clarification if something was unclear. The instruction was not to solve the case quickly but to ponder and collaborate through interactive discussion to learn the skills of interprofessional work.

**Fig. 1:**
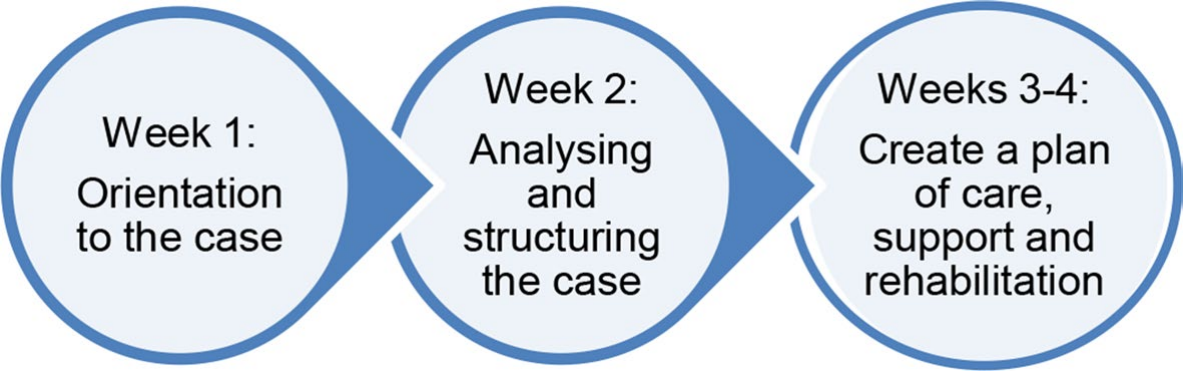
Describes the three different phases of the four-week collaborative online case assignment

The case assignment was structured to unfold in three phases (Fig. 1). To initiate discussion, the course coordinator offered three to five guiding questions such as “Which aspects capture your attention?” or “Which questions are most central at this phase of the evaluation?”, at the beginning of weeks 1 and 2. In the third week, also new case information was provided to support successful task construction and solutions. After each phase the groups wrote conclusions of observations, plans or evaluations using wiki-tool. The texts produced in wiki were not analysed, since the intention was to study the occurrence of MR and SSMR during the collaborative learning.

Every group was assigned a designated teacher to oversee the discussion and provide support, but the students had sole responsibility for completing the task week by week. Each group could also access expert consultation, including participating professionals from medicine, occupational therapy, and psychology. The whole course was as evaluated pass/fail.

#### Coding of the data

The data comprised written discussion comments from the small interprofessional groups; the comments of coordinators, teachers, and consultants were excluded from the analysis. This decision was made to focus namely on the student-led, group learning interactions (see Volet et al., 2009) and since teachers´ role was more to oversee the discussion. Moreover, the teacher facilitative structure had not fully taken form during the pilot stage of the course. Coding proceeded as a phased analysis of MR and SSMR; participants’ comments were pseudonymized at the outset. The number of comments produced varied across groups A–G, ranging from 55 (A) to 192 (F); in total, 896 comments were analysed, based on 6202 sentences (see Table 1). As comments varied in length (from 1 to 45 sentences), they could not be used as the unit of analysis; instead, it was decided to count all produced sentences and to use the sentence as the main unit of analysis. A sentence was defined as a combination of words that typically begins with a capital letter and ends with a period or a question mark.

**Table 1:** Coded data from the small-group discussions

	A	B	C	D	E	F	G	In total	Mean
All coded data No. of comments	55	108	123	187	131	192	100	896	128
No. of sentences in comments (including MR-sentences)	557	806	916	1031	922	1368	602	6202	886

The next step was very demanding: to identify instances of MR in the comments and separate these from the cognitive activity of knowledge construction (e.g., adding information). The given case assignment was not explicit, instead the groups needed some creativity and imagination to solve the patient cases. For that reason, valid MR coding demanded a lot of time and effort. After multiple re-readings, instances of MR sentences were identified—ideas, thoughts, questions, or explanations of varying length that aimed to regulate the group´s joint learning processes or group members´ cognition (or oneself as a group member). The comments were usually comprehensive, thereby creating meaningful context to MR sentences. Examples of short MR sentences can be illustrated by the following: “What is our doctor´s decision?” or “Would at least (student´s name) have tools for this?”. If there was insufficient evidence for MR or SSMR, it was coded as not having MR or SSMR. A lengthy, MR sentence could appear as a continuous sentence or as a progression of sentences such as, “We cannot go forward with discussion if we don´t “come up” that we know facts about the person? Since we don´t yet have more information about the case. It all rests on our evaluation at this point”. More examples of MR can be found in Table 2.

Some of the candidate sentences clearly addressed guiding questions and could not therefore be classified as MR. For example, one answer to the guiding question “What knowledge do you need more of?” was “I would like to discuss with the wife and know what the customer has done before the accident…this information would be helpful later in planning the rehabilitation”; this was not defined as MR.

Following this phase of data processing, it was decided that MR should address regulative efforts involving another group member/members or the whole group. Accordingly, MR was analysed only at group level, and where an individual stated that the group had impacted his/her cognition, this was coded as MR. Instances of knowledge construction (sentences that added or elaborated information) were among the produced sentences but also omitted from MR (see examples in Table 2),as were sentences copied in someone else’s words from another comment or from the internet. Finally, off-task discussion (which was almost nonexistent) was not coded. 

**Fig. 2:**
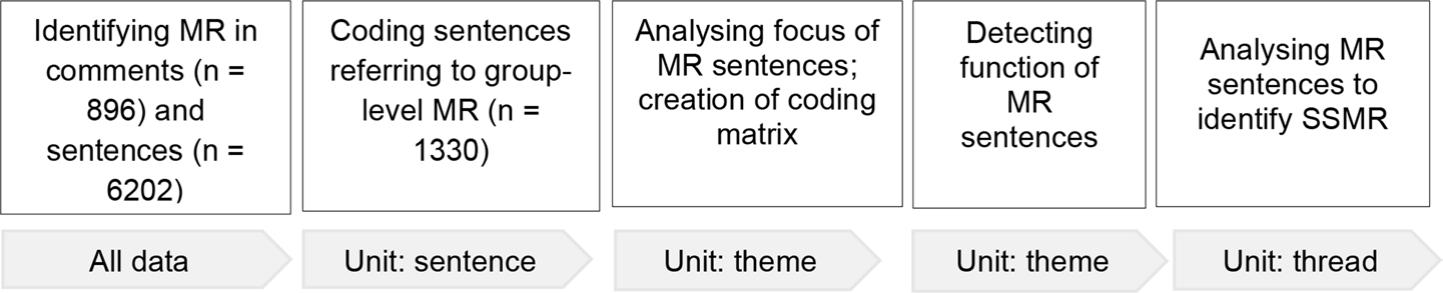
Demonstrates how coding the data progressed in stepwise manner from the identification of metacognitive regulation from all data to applying three units of analysis as sentence, theme and thread

When analysing the content of online asynchronous group discussions, the unit of analysis may vary within a single study (De Wever et al., 2006). In the present case, the unit of analysis was tripartite: sentence-level, thematic (focus and function), and thread-level (identified as SSMR) (see Fig. 2).

### Coding of MR focus and function

After identifying the emergent MR sentences, we performed a thematic content analysis and identified a dominant focal theme for each. The focus of MR could relate to one sentence or more—for example, *a sentence involving asking* followed by *a sentence involving reasoning or argument*. This stage of analysis was partly theory-driven and applied Khosa and Volet’s (2014) distinction between two qualitatively different levels of regulation: MR as low-level task production (theme 1) and MR as high-level content resolution (theme 2). Upon further analysis, inductive coding revealed two additional themes; content of interprofessional aspects (theme 3) and group collaboration (theme 4). The third round of coding included detecting the function of each MR sentence (i.e., planning, monitoring, or evaluation) according to previous metacognitive research (Schraw & Moshman, 1995). Examples of the different coding categories for MR and SSMR can be found in Table 2.

#### Coding of SSMR

Finally, each group discussion was coded in terms of the emergence of SSMR. To identify this shared process while preserving the dynamics of interaction, thematic units (focus) were analysed at thread level. As MR sentences need not follow one after another (Iiskala et al., 2015), coding may relate to individual turns (comments, sentences) and to group-level threads (Volet et al., 2013). As SSMR refers to social learning processes, an SSMR thread must include at least two individual turns regulating the same cognitive matter.

**Table 2:** MR and SSMR coding: definitions and examples

	Definition	Example
Not identified as MR or group-level MR	A student adds information or viewpoints for patient case co-construction. A student regulates individual thinking processes with no clear reference to the cognitive efforts of the group or others (Flavell, 1976).	*“Since the child has multiple difficulties in widely different areas*,* an individual neuropsychological study could be done. First I would do WISC-IV test…”.* (1P) *”That loneliness is a big deal. Is she depressed*,* mourning her husband. However*,* that is quite a recent event.”* (34F)
Focus of MR	Four themes: 1. Task production 2. Content; case-related 3. Content; interprofessional coordination 4. Group collaboration	**1.Task production** (low-level MR) “*Let’s all remember to answer to this message that 15S started so that the message chain remains clear*” (28L). Function: monitoring “*Tomorrow we should apparently provide some kind of summary of our work. Who does this? I would like to suggest…”* (17S), Function: planning
Low-level and high-level MR	Task production as low-level MR: Students regulate group learning of practical information related to the assignment. Content level as high-level MR: Students regulate group learning involving understanding, explanation, and justification related to resolving the case (Volet et al., 2009; Khosa & Volet, 2014)	**2. Content addressing case assignment** (high-level MR) “*I feel there is some kind of problem with the boy´s behaviour…I don´t know…What do you others think?”* (19S), Function: monitoring “*I also agree that it is very important to evaluate now and not (to wait) until the nursing home*,* as we can then evaluate what kind of living Tapani (name) could be settled.”* (32LL) Function: monitoring
Function of MR	Planning, monitoring, evaluating (Schraw & Moshman, 1995)	**3. Content addressing interprofessional aspects** (high-level MR) “*I would also like to know about methods; do you 28L have to screen the level of language? I wonder if there are some possible evaluations that overlap and whether we can take these into consideration as we plan the test battery.”* (40P), Function: monitoring *“Would it make sense if everyone first wrote down the central questions and methods of evaluation from their professional point of view*,* and then we can prioritize them and identify overlaps? in that what evaluations could be done interprofessionally?” (47T)*, Function: planning **4. Group collaboration** “45T *has already presented the occupational therapist’s point of view very well*,* and others have also come up with a lot of things (content) that I would not have immediately thought of. I think we already have a very solid start! I would stress that we have a patient with a memory disorder that we should examine…”* (21T). Functions: evaluating, monitoring “*I’m also thinking about the day center issue*,* mostly from the point of follow-up. If any of the teachers monitor this conversation*,* could someone explain to us what is meant by ‘the day center?’” (46T)* Function: monitoring missing knowledge and group understanding
Socially shared metacognitive regulation (SSMR)	SSMR occurs when the learning process engages at least two students in regulatory activities who in turn interdependently affect the course of the group’s cognitive learning process (Iiskala et al., 2004, 2015; Volet et al., 2013).	**SSMR thread involving four students** “*I´m a bit surprised how strongly social work has been taken into account in all of our texts*” (15S), Function: evaluating \ *“I think these issues are not just social work-related…I believe that*,* as professionals who are part of this assignment*,* we all have a duty to think primarily about what´s best for the client and to promote this via each profession”* (24T), Function: evaluating \ *“As a general comment on 15S and 24T’s discussion about interprofessional work and views*,* I think it´s great that we have such a range of professional skills and views*,* and I feel that trusting in other professionals liberates resources to concentrate one´s own competence” (40P).* Function: evaluating \ *“Anyhow*,* it has been great to see how the other professions also view the wider situation and not just their own specific area separated from the wider context” (12S)*, Function: evaluating

### Inter-Rater reliability

MR sentences, MR focus, MR function, and SSMR were coded by the first author and co-coded by the third author for inter-rater reliability. Coding was described and taught in each of the four stages. First, the coding was rehearsed using test data. Next, the co-coder randomly chose two groups to code, comprising 25% of the comments (1518 sentences resulting 336 MR sentences). Values for percentage agreement and inter-rater agreement are shown in Table 3. Inter-rater reliability was assessed using Gwet´s AC1 statistic measurement in stages 1 (MR) and 4 (SSMR). These stages involved binary categories (presence of MR/SSMR or absence of MR/SSMR). Gwet´s AC1 adjusts better for chance agreement and the analysis yielded substantial agreement between raters (see Wongpakaran et al., 2013). Cohen´s kappa was applied to estimate inter-rater agreement in stages 2 and 3 (MR focus and function), indicating substantial kappa values for MR focus and moderate for MR function (see Landis & Koch, 1977, p. 165). The majority of disagreements related to issues such as individual consultations that were sometimes regarded as utterances that benefited the whole group (i.e., as group-level MR). At each stage of inter-rater coding, disagreements were discussed and resolved.

**Table 3:** Inter-rater agreement on coding (four stages)

Coded data (*n* = 1518 sentences, 336 MR sentences)	Total agreement %	Gwet´s AC1 and Cohen´s kappa
1. MR sentences	81.9	0.736
2. MR focus	75.3	0.636
3. MR function	85.4	0.589
4. SSMR	84.8	0.796

## Results

### Emergence of metacognitive regulation (MR)

We identified the emergence of MR in all seven of the interprofessional group discussions. In total, 1330 MR sentences were produced. The detected group differences in percentage emergence of MR ranged from 15.3% (group A) to 25.1% (group B); the mean for all groups was 21.5%. In three groups (A, F, and G), the incidence of MR was less than the mean score. Group MR increased with the number of sentences produced, with the exception of group F (see Table 4). In addition, z-testing revealed significant statistical differences across all groups (chi2(6) = 34.69; *p* < .001; *V* = 0.08). These results indicate that IPL and collaboration levels vary; some groups seem to be at risk of failing to achieve collaborative learning beyond the addition of information.

Table 4 summarizes all the coded data from the seven groups and shows the distribution (%) of emergent MR, focus, function, level, and SSMR. The results are further explained in relation to each research question.

**Table 4:** Summary of coded data: emergent MR and focus, level, and function of MR and SSMR

Group								
	A	B	C	D	E	F	G	Mean
**1. Emergent MR: all produced sentences (%)**	15.3	25.1	23.1	24.7	21.8	18.4	20.6	21.5
**2. Focus of MR (%)**								
*Task production* (low-level)	*18.8*	*8.9*	*8.0*	*23.5*	*28.4*	*18.0*	*25.0*	18.4
*Content resolution* (high-level)	*56.5*	*83.7*	*81.6*	*57.3*	*66.2*	*64.0*	*49.2*	66.8
- The case	(41.2)	(43.1)	(58.5)	(30.6)	(47.3)	(48.6)	(41.1)	(44.3)
- Interprofessional coordination	(15.3)	(40.6)	(23.1)	(26.7)	(18.9)	(15.1)	(8.1)	(21.1)
*Group collaboration*	*24.7*	*7.4*	*10.4*	*19.2*	*5.5*	*17.1*	*25.8*	13.5
*100%*								
**Function of MR (%)**								
- Planning	4.7	3.0	4.3	3.9	4.0	7.0	8.1	5.0
- Monitoring	80.0	88.1	86.8	75.7	91.0	88.0	75.0	83.6
- Evaluating	15.3	8.9	8.9	20.4	5.0	5.0	16.9	11.3
100%								
**3. SSMR in MR sentences produced (%)**	75.3	90.6	84.9	87.5	95.0	89.2	75.8	85.5

### Focus, function, and level of emergent MR

Next, looking at the focus and function of emergent MR sentences, the results show a focus on four distinctive themes in every group: task production, content (case-related), content (interprofessional coordination), and group collaboration. The identified categories of MR (task production and content-level regulation) align with Khosa and Volet (2014). However, beyond resolving the case, the distinctive element in this learning environment (and hence the data) was to learn about interprofessional coordination and integration. For that reason, the focus of content regulation was necessarily divided between general case solving and interprofessional solving. In addition, we found that groups regulated their own collaborative learning as participants observed how learning was progressing in terms of collaborative effort and level of shared knowledge. To that end, they sought to benefit from the expertise and knowledge of other group members —for example, to enhance everyone’s understanding of a medical concept or test result—and this emerged as the fourth theme. Table 5 describes the focus of MR in terms of the four themes.

**Table 5:** MR focus: four themes (with descriptions)

1. Task production (low-level MR)	2. Content: case-related (high-level MR)	3. Content: interprofessional (high-level MR)	4. Group collaboration
Regulation of the production and organisation of the case assignment	Regulation of the general case content of the assignment	Regulation of the interprofessional content of the assignment	Regulation of the group’s collaborative learning
Descriptions			
- Coordination of writing - Regulation of time taken to complete the assignment - Criticism of information provided for assignment	- Attempting to understand the given information - Evaluating realistic solutions for a given case	Discussion of coordination, division of work, and overlapping professional fields (e.g., testing)	- Evaluation of group’s collaborative effort in reaching outcomes - Monitoring collaboration difficulties

The task production focus accounted for between 8.0% and 28.4% (*M* = 18.4) of group discussions related to the regulation of low-level cognitive activities. On the other hand, high-level content regulation accounted for between 49.2% and 83.7% (*M* = 66.8), indicating a greater focus on high-level cognitive activities. Regulation related to the fourth theme (group collaboration) accounted for between 5.5% and 25.8% of group discussions. (For an overview, see Table 5).

Using z-tests, the statistical analysis of these thematic aspects of MR focus revealed the following significant differences among the groups. *Task production*: chi2(6) = 48.83; *p* < .001; *V* = 0.19; *content (case-related)*: chi2(6) = 40.24; *p* < .001; *V* = 0.14; *content (interprofessional)*: chi2(6) = 67.34; *p* < .001; *V* = 0.23; g*roup collaboration*: chi2(6) = 50.14; *p* < .001; *V* = 0.19.

High-level content-related MR (themes 2 and 3) also differed significantly across the groups (chi2(6) = 79.97; *p* < .001; *V* = 0.25). Among these variations in MR focus, the third theme (interprofessional content) is of particular interest as one of the core elements of IPL. More specifically, the group differences indicate versatile focus on the regulation of interprofessional coordination and integration.

**Fig. 3:**
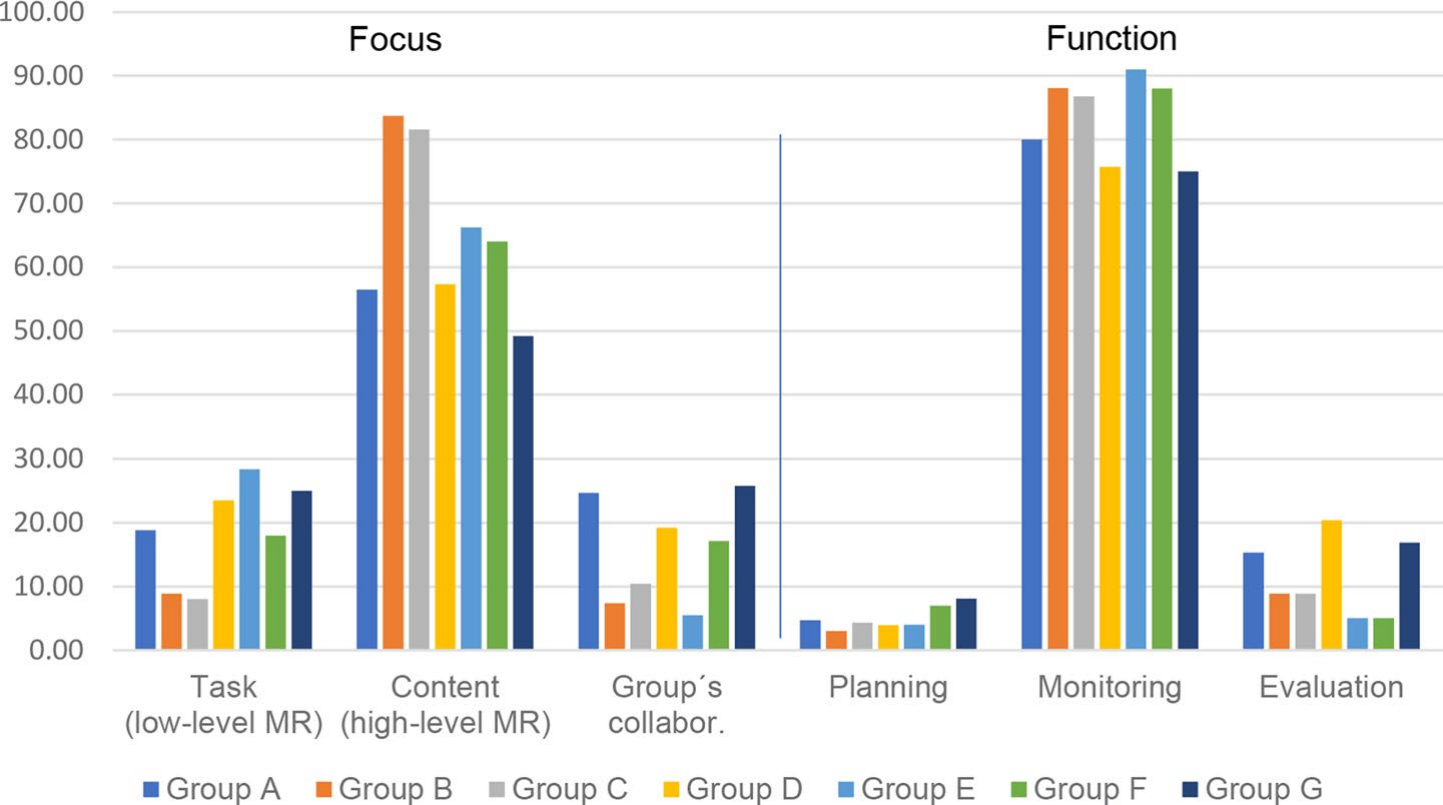
The bar chart demonstrates the percentage distribution of focus and function of metacognitive regulation across the seven groups (named **A**-**G**). The focus as themes 2 and 3 are combined to represent high-level content regulation. In every group the discussion includes more high-level than low-level metacognitive regulation. Similarly, the main function is monitoring across the groups

The functions of MR (planning, monitoring, and evaluating group cognitive processes) were similarly distributed across the groups. The results identify *monitoring* as the major function (75.0–91.0%, *M* = 83.6%); less attention was devoted to *planning* (3.0–8.1%, *M* = 5.0%) or *evaluating* (5.0–20.4%, *M* = 11.3%). There were significant statistical differences across the groups in terms of monitoring (chi2(6) = 6.68; *p* = .352; *V* = 0.07) and evaluating (chi2(6) = 48.57; *p* < .001; *V* = 0.19) but not in relation to planning. This indicates that all the interprofessional groups spent a similarly brief amount of time regulating planning during collaborative learning; that is, there was little discussion of issues like committing to, or reaching learning goals. Figure 3 shows the percentage distribution of MR focus and function in the seven groups (A–G); themes 2 and 3 are combined to represent high-level content regulation.

**Fig. 4:**
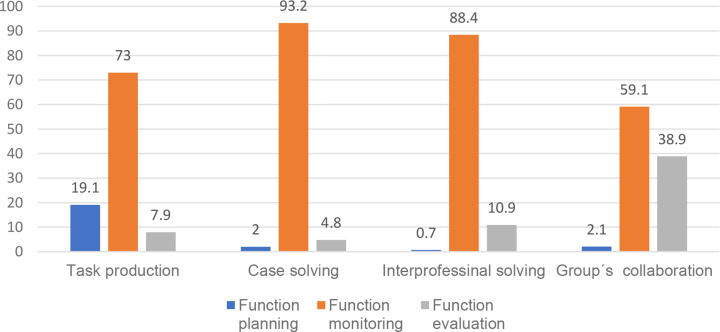
The bar chart shows the associations (%) between function and focus of metacognitive regulation (all groups). The connection was significant, with a p value of < 0.001. The figure shows how the planning function is linked to task production and group collaboration was most strongly linked to the evaluative function

Using crosstabulation, we also analysed the relationship between MR focus (task production, case-related content, interprofessional content, and group collaboration) and MR function (planning, monitoring, evaluating) and found a statistically significant relationship (c^2^(6) = 299.1; *p* < .001; *V* = 0.336). The analysis indicates that the planning function is linked to task production. The level of monitoring was high across the four themes (especially for themes 2 and 3), and group collaboration was most strongly linked to the evaluative function (see Fig. 4).

### Emergence of SSMR

Finally, regarding the third research question, we wanted to know to what extent MR was socially shared. We found that 75.3% or more of each group’s MR sentences involved SSMR; in two groups, the incidence of SSMR was greater than 90%. Here too, *z* tests revealed statistical differences between the groups (chi2(6) = 40.12; *p* < .001; *V* = 0.17; in short, although much MR was socially shared, this clearly varied across the groups. Table 5 provides a summary of SSMR coding.

## Discussion

The qualitative design of the present study “zoomed in” on interprofessional collaborative learning and examined cognitive regulation in small groups. The study traces the emergence of MR and SSMR and the components of MR focus and function. Linking these findings to previous research, we can derive some useful theoretical insights and practical implications.

Regarding MR emergence, we found that MR accounted for 20–30% of university students’ collaborative learning, which aligns with Khosa and Volet (2014) and Iiskala et al. (2021). Further analysis revealed significant differences between our small interprofessional groups, indicating that MR should be supported to ensure that collaborative learning extends beyond the provision of additional information. In short, interprofessional collaboration must aim for knowledge integration, and MR plays a critical role in this regard. To reinforce interprofessional dialogue and integration, Mönkkönen and Kekoni (2023) stressed the role of questioning as a central discussion tool for sharing, understanding, and testing the case at hand. Similarly, Vauras et al. (2003, 2009a) noted that open questions and tentative statements appear to foster mutual problem solving and promote high-level regulation.

To gain a deeper understanding of MR during interprofessional collaborative learning, we then investigated the focus and function of emergent MR. Surprisingly, we found that MR extended beyond task production to two distinct forms of content regulation (case-related and interprofessional), along with a focus on regulating group collaboration. While the distinction between low-level (task production) and high-level (content) regulation aligns with Khosa and Volet (2014), we also found a dominant focus on high-level (case-related and interprofessional) content rather than on low-level task production. In other words, our students’ collaborative learning involved more regulation of the assigned case rather than task organisation or assembly. In this way, the students were able to regulate their collaborative understanding rather than merely completing the task at a more superficial level. This is a highly relevant skill for interprofessional working life and indicates that the learning process is genuinely collaborative. In light of the clear quantitative differences between the groups in this regard, content-level learning should be deliberately facilitated during IPL.

Interestingly, the regulation of interprofessional content coordination and integration varied across the groups (8.1–40.6%). For example, when comparing the groups that are most distant from one another in more detail (strongest group B, 40.6% and weakest group G, 8.1%), an increased number of contrasting positions were observed between these groups. The group B, focused also strongest on overall content regulation (83.7%), less on the low-level task regulation (8.9%) and further, had the highest emergence of MR sentences (25.1%) of all groups. On the other hand, the group G, focused the least on the overall content regulation (49.2%), high on low-level task regulation (25.0%) and had below average of emerged MR (20.6%). Interestingly, their focus on collaborative regulation was the strongest of all groups (25.8%), while group B regulated their collaboration only with 7.4%. The factors contributing in variation in regulation of interprofessional content coordination needs further investigation in the future. Nevertheless, the considerable variation suggests that the key learning goal of interprofessional collaboration may be jeopardized by inadequate attention to interprofessional aspects. Issues like professional role blurring and boundary confusion can be seen as barriers to effective collaboration (Hall, 2005; Suter et al., 2009). On the other hand, the need to negotiate around integration and overlaps can be seen as a positive learning opportunity to share and understand each other´s “cognitive maps” and break down unnecessary silo effects (Hall, 2005). According to Vauras et al. (2003), negotiation is an important element of collaborative knowledge creation and requires space to unfold.

Our findings confirm that the regulation of group collaborative learning (the fourth theme) is a key aspect of IPL—that is, observing knowledge gains and understanding knowledge deficits or a lack of clarity that might require further consultation. They evaluated the collaborative progress and the interprofessional benefits of learning together. The results suggest that the participating higher education students were able to adopt a “bird’s-eye view” in monitoring their joint efforts to achieve genuine collaborative learning and the desired learning outcomes.

Regarding the functions of MR, our results again align with Khosa and Volet (2014), as monitoring predominated in every group at the expense of planning. Indeed, the lack of planning may have reinforced the need for monitoring (Iiskala et al., 2015). On tracing the links between MR focus and function, we found that planning was most often associated with a focus on task production, possibly because the week-by-week unfolding of the course assignment meant revisiting the task to plan for phased completion. Similarly, the evaluative function was linked to group collaboration. In general, planning and evaluation must be regulated to ensure collaborative learning outcomes (Järvelä et al., 2016b) and should therefore be highlighted by supporting these regulatory functions explicitly and consistently throughout the learning process.

Turning to the final aim of the present study, we found that MR was largely reciprocal meaning MR was shared between the participants in every group (75%+). Our results align with Iiskala et al. (2021), who reported levels of 85–92% SSMR in university students’ collaborative discussions. In addition, we know that resolving complex cases can trigger SSMR (Iiskala et al., 2011). However, as previous studies also indicate some variations in student SSMR (De Backer et al., 2020; Iiskala et al., 2015), health and social care student engagement in MR and SSMR during IPL warrants further research.

### Limitations

The present study has some limitations. First, contrary to course guidance, some discussions may have occurred beyond the electronic platform. The online and synchronic interprofessional collaborative learning has its benefits, still asynchronic learning can be discouraging some students (see Borg et al., 2021). Also, the assigned clinical cases were intended to be similarly demanding. However, topic-specific differences may have affected the groups´ regulation. Additionally, small groups´ professional composition could have an effect on the groups´ regulatory activities. Future research should further explore these issues. In addition, the creative nature of the case assignment may have affected the coding of MR functions; for example, a decision was made to code MR sentences that contributed to case-related content solving as *monitoring*. However, collaborative creation of content could also indicate *planning* of collaborative effort toward accomplishing the task. Additionally, as the coding of MR already acknowledged only group-level regulative sentences, this may have increased the incidence of SSMR. This study focused namely on students´ collaborative interaction as they practiced interprofessional work together. We know that the teacher facilitation varied across groups, and this may have affected group learning processes and the emergence and level of MR and SSMR, hence might bias the results. The impact of teacher facilitation should be analysed in future research. As the assignments did not support singular correct solutions, it was not possible to analyse the links between MR and group solutions to determine how learning outcomes were reached. The connection between groups´ collaboration and learning outcomes warrants further investigation.

These results offer some valuable theoretical insights into the role of MR in interprofessional collaborative learning. By exploring MR emergence, focus, and function and the role of SSMR, we identified some supports that may enhance IPL and ensure high-level collaborative learning.

### Implications for educational practice: enhancing and facilitating MR and SSMR

Even in student-led IPL learning situations, where the main aim is specifically to practice interprofessional collaboration, the teacher´s support is valuable. Based on our results and previous literature there are some practical implications for enhancing and supporting emergence of MR and SSMR in IPE.

When building interprofessional learning environments, educators should ensure that the chosen methods, assignments, and facilitation strategies support student groups´ high-level collaborative learning. According to Smeets et al. (2024), students must receive pre-collaboration preparation to promote interdependence and interaction. Unfortunately, Fox et al.’s (2017) review reported that interprofessional students do not usually receive teamworking guidance prior to group activity. In the present case, while students were given some guidelines for collaborative discussion, that guidance was fairly minimal. More explicit guidelines of interprofessional collaborative learning, both in online or real-world face-to-face interaction, could help groups to understand how to regulate their joint effort and sustain high-level collaborative learning processes.

In addition to guidelines, it is important to consider the facilitation techniques. In a recent study of interprofessional learning, Bogossian et al. (2023) reported that the chosen learning methods proved less collaborative than the educators intended. One practical implication is that due attention must be paid to facilitative teaching techniques. In their review of teachers’ facilitation strategies for online IPE, Evans and Perry (2023) identified a range of techniques used to support group discussion, including direct instruction, amplifying student posts, encouraging shared reflection and modeling to enhance collaborative interaction.

While the role of teacher facilitation is important, students must also be afforded time and opportunities for regulation. The study of Lorello et al.’s (2024) findings indicate that medical supervisors may have disrupted student self-regulation by taking control of the learning situation. To facilitate group´s cognitive regulation, teachers should allocate sufficient time for discussion and questioning without providing direct answers (see Van Diggele et al., 2020). Consistent with the studies of Volet et al. (2009); Iiskala et al. (2021), this study found that university students are capable to metacognitively regulate if provided sufficient time and possibilities.

According to Wilhelmsson et al. (2012, p. 90), “simply letting students work in teams” does not suffice to ensure genuine and productive collaborative learning. For example, the findings of this study show that each small group allocated only limited time on regulatory function of planning. Using techniques like question prompts (Järvelä et al., 2016a; Zion et al., 2015), teachers can encourage student groups to regulate their assignments through collaborative learning. Relevant prompts might include the following questions: “Have we planned collaboratively how to reach the learning goals?”; “Have we considered everyone´s views and gained from each others´ knowledge?”; and “Are we on the right path to complete this assignment?” To further enhance metacognitive planning, monitoring, and evaluation, Biasutti and Frate’s (2018) Group Metacognition Scale (GMS) could be deployed to measure metacognitive skills and identify any strengths and weaknesses related to group collaboration.

It seems possible, then, to teach and support MR (see also Järvelä & Hadwin, 2013; Schraw, 1988), and every interprofessional group should have the tools to achieve high-level collaborative learning. However, we lack explicit guidance for supporting the emergence of MR and SSMR during interprofessional collaborative learning. Based on the results of the present study, Box 1 summarizes the key implications for both teachers and students seeking to enhance and support MR and SSMR in IPL settings.

**Box 1:** Key implications: How both teachers and students can enhance group-level MR and SSMR in IPL settings

• Explicate and recognise the aims of collaborative IPL (e.g., what it means; what it aims for). • Know the means by which a group can metacognitively regulate collaborative learning
○ The importance of high-level content learning (understanding, critical thinking, problem solving, justification, interprofessional integration). ○ Awareness and ability to use MR functions (planning, monitoring, evaluation) in a balanced way throughout collaborative learning. ○ Allocate sufficient time for MR and SSMR to unfold.
• Recognise the importance of negotiating professional knowledge, views, and methods and addressing overlapping issues) • Know the importance to share and make MR reciprocal between participants (through SSMR).

## Conclusion

In interprofessional collaborative learning settings, the aspects of MR and SSMR explored here enhance student learning and knowledge acquisition and offer teachers and educators the necessary tools to support high-level learning processes and the pursuit of desired IPL outcomes. Understanding the role of regulation in collaborative settings seems likely to enhance future collaborative practices in working life, but the present findings indicate notable differences in how small interprofessional groups regulate their collaborative learning. In some groups, where collaboration is barely regulated and interprofessional views are not integrated, learning consists mainly of providing each other with additional information. In such cases, there is a real risk that the benefits of IPL may not be fully realized during collaborative learning. For that reason, the role and importance of MR and SSMR should be highlighted and more explicitly supported in interprofessional collaborative learning contexts. Our results indicate that health and social care professional students need to develop a greater awareness of these regulatory processes if they are to achieve the desired collaborative learning goals. Explicating the aim of collaborative learning and facilitating MR and SSMR can enhance students’ ability to work in interprofessional group and team settings. Understanding what triggers MR in collaborative learning contexts and how individual regulation and teacher facilitation impacts a group’s collaborative learning are central issues for future research.

## Data Availability

Data presented in this study are available on reasonable request from the corresponding author.
